# PPARα-dependent Insig2a overexpression inhibits SREBP-1c processing during fasting

**DOI:** 10.1038/s41598-017-10523-7

**Published:** 2017-08-30

**Authors:** Jae-Ho Lee, Hye Suk Kang, Hyeon Young Park, Young-Ah Moon, Yu Na Kang, Byung-Chul Oh, Dae-Kyu Song, Jae-Hoon Bae, Seung-Soon Im

**Affiliations:** 1 Department of Physiology, Keimyung University School of Medicine, Daegu, 42601 South Korea; 2 Department of Molecular Medicine, Inha University School of Medicine, Incheon, 22212 South Korea; 3 Department of Pathology, Keimyung University School of Medicine, Daegu, 42601 South Korea; 4 Lee Gil Ya Cancer and Diabetes Institute, College of Medicine, Gachon University, Incheon, 21999, Korea; Department of Physiology, College of Medicine, Gachon University, Incheon, 21999 South Korea

## Abstract

Peroxisome-proliferator-activated receptor alpha (PPARα) and sterol regulatory element-binding protein (SREBP) play a role in regulating cellular fatty acid and cholesterol homeostasis via fatty acid oxidation and lipogenesis. The control of SREBP processing is regulated by the insulin induced gene (INSIG)2a protein, which binds SREBP to prevent SREBP translocation to the Golgi apparatus during nutrient starvation in the liver. However, the regulation of SREBP-1c processing by INSIGs during fasting and the regulatory mechanisms of the mouse *Insig*2*a* gene expression have not been clearly addressed. In the present study, we found that *Insig2a* was upregulated by PPARα in mouse livers and primary hepatocytes during fasting, whereas *Insig2a* mRNA expression was decreased in the livers of refed mice. A PPAR-responsive element between −126 bp and −114 bp in the *Insig2a* promoter was identified by a transient transfection assay and a chromatin immunoprecipitation assay; its role in regulation by PPARα was characterised using *Pparα*-null mice. These results suggest that PPARα is a trans-acting factor that enhances *Insig2a* gene expression, thereby suppressing SREBP-1c processing during fasting.

## Introduction

Sterol regulatory element-binding proteins (SREBPs), including SREBP-1a, SREPB-1c, and SREBP-2, are major transcription factors that regulate fatty acid and cholesterol synthesis. They are localised to the ER membrane as inactive precursors and are tightly associated with the SREBP cleavage-activating protein (SCAP)^1^. SCAP also interacts with insulin-induced gene (INSIG) proteins to retain the SCAP/SREBP complex in the ER. When cellular cholesterol levels are low, the SCAP/SREBP complex dissociates from INSIGs and moves to the Golgi apparatus, where proteolytic cleavage occurs and the N-terminal transcription factor domain of SREBPs is released. The cleaved SREBPs enter the nucleus, where they activate the transcription of target genes. Among the SREBP isoforms, SREBP-1c primarily regulates genes involved in fatty acid and triglyceride (TG) synthesis; its mRNA and protein levels are mainly regulated by insulin^2^.

INSIGs have crucial roles as regulators of SREBP processing; they bind SCAP to prevent translocation of the SCAP/SREBP complex to the Golgi apparatus^3^. In mice, there are three types of *Insig* mRNAs (*Insig1, 2a*, and *2b*) that encode two INSIG isoforms: INSIG1 and INSIG2. The regulatory roles of INSIGs on SREBP processing and their effects on liver lipid metabolism have been demonstrated in mouse models. Disruption of both *Insig1* and *Insig2* in the mouse liver results in an excessive build-up of cholesterol and TGs in the liver because of the continuous activation of SREBP-1 and SREBP-2^4^. Expression of *Insig1* and *Insig2* is reciprocally regulated in the mouse liver^5^. Expression of *Insig1* is upregulated by feeding, while the expression of *Insig2a*—the predominant form of *Insig2* in the liver—is decreased by feeding but elevated upon fasting or through glucocorticoids^5–7^. However, the molecular mechanisms of the transcriptional regulation of *Insig2a* in fed and fasting states are not completely understood.

Peroxisome proliferator-activated receptor alpha (PPARα) is a nuclear receptor that is expressed in the liver, brown adipose tissue, heart, and kidney^8^. PPAR plays an essential role in homeostasis during nutritional deprivation by regulating the expression of genes required for fatty acid uptake and oxidation, TG hydrolysis, ketogenesis, and gluconeogenesis^9–11^. The roles of PPARα in different metabolic conditions have been elucidated using *Pparα*-null mice. During fasting, *Pparα*-null mice exhibited pathological phenotypes such as severe hypoglycaemia, hypoketonaemia, hypothermia, and elevated plasma free fatty acid levels^11^. A chronic high-fat diet led to severe hepatic steatosis in *Pparα*-null mice^12^. These phenotypes imply that PPARα has an essential role in the regulation of fatty acid uptake and oxidation. Although the direct targets of PPARα have been identified by chromatin immunoprecipitation (ChIP)-Seq analyses, the functional relevance and regulatory mechanisms of the target genes of PPARα have not yet been elucidated^13^.

Here, we report that *Insig2a* is regulated by PPARα through a PPAR response element (PPRE) in the promoter region of the *Insig2a* gene. This suggests that PPARα ligands could be promising targets for combatting hepatic steatosis by repressing lipogenesis and hyperlipidaemia through increasing *Insig2a* gene expression, followed by the inhibition of SREBPs.

## Results

### *Insig2a* is upregulated in the livers of fasted mice

**Figure 1:**
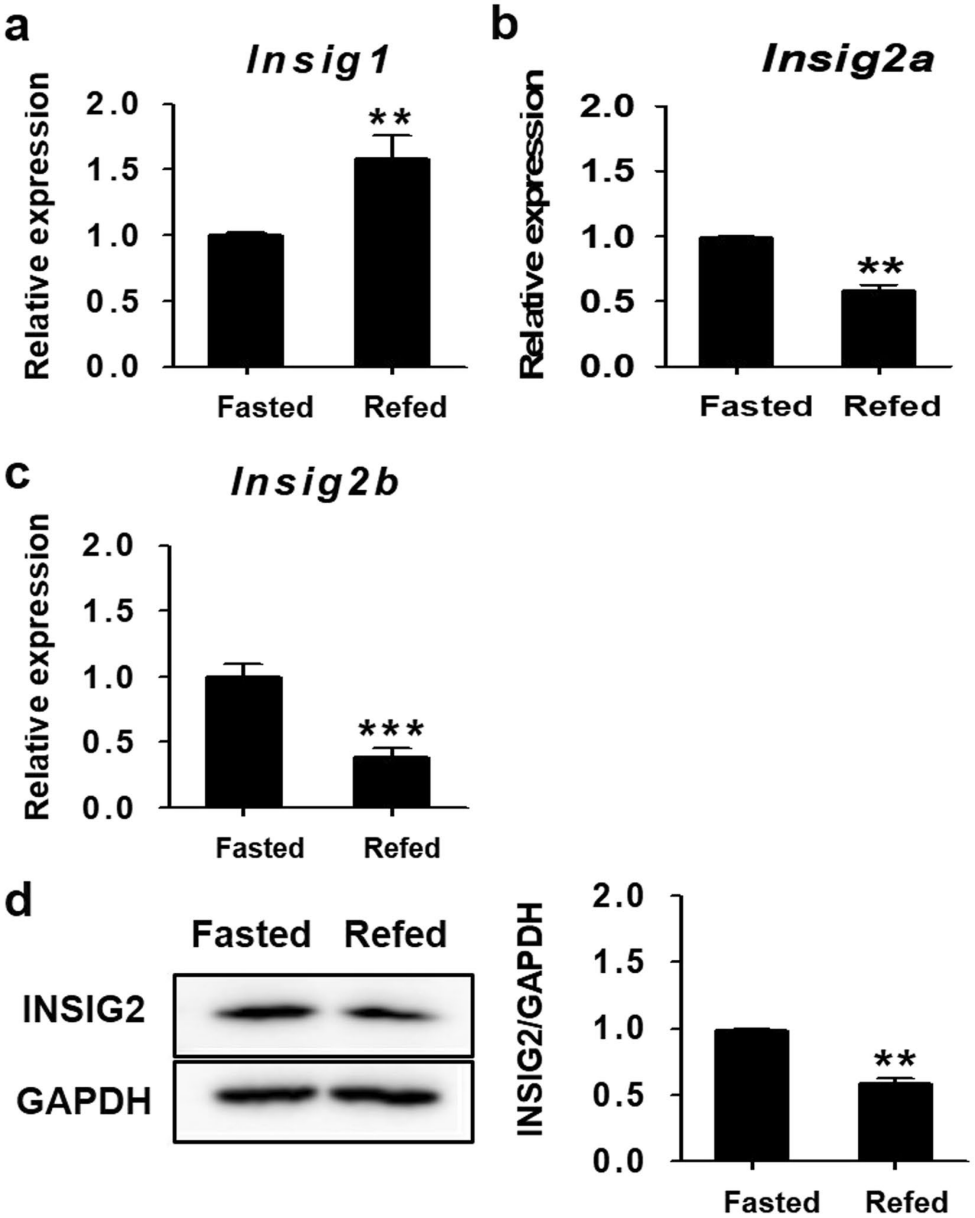
Fasting elevates *Insig2a* gene expression. In the livers of wild-type (WT) mice that were fasted for 24 h (fasted) or refed for 12 h after 24 h fasting (refed), mRNA expression levels of *Insig1* (**a**), *Insig2a* (**b**), and *Insig2b* (**c**) were analysed by qPCR analysis. The expression levels of these genes under fasting conditions were regarded as 1.0. (**d**) Protein levels of INSIG2 in the livers of fasted and refed WT mice. *Right panel*: Densitometry calculations for western blot data. INSIG2 induction levels were quantified using ImageJ software and normalised by GAPDH (cropped; full length blots can be found in Supplementary Fig. S1). **p < 0.01 and ***p < 0.001 vs. refed mice.

In previous gene expression profiles using microarray analysis in the livers of fasted and refed mice, hepatic *Insig1* gene expression was upregulated by refeeding. Conversely, *Insig2* expression was lower in refed livers than in fasted livers^14^. To confirm the expression levels of *Insig* genes in the livers of fasted and refed mice, the changes in *Insig* gene expression were verified by reverse transcriptase quantitative PCR (RT-qPCR) analysis. The mRNA level of *Insig1* showed a 1.5-fold increase in the refed mice compared with it in the fasted mice (Fig. 1a). On the other hand, the mRNA level of *Insig2a* was higher in fasted mice than in refed mice (Fig. 1b). *Insig2b* mRNA expression was also higher during fasting than after refeeding (Fig. 1c). Similarly, INSIG2 protein activity was increased in fasted mice livers (Fig. 1d). These results confirmed that *Insig2a* is upregulated by fasting at both the mRNA and protein levels, whereas its expression was downregulated by refeeding.

### PPARα upregulates *Insig2a* during fasting

**Figure 2:**
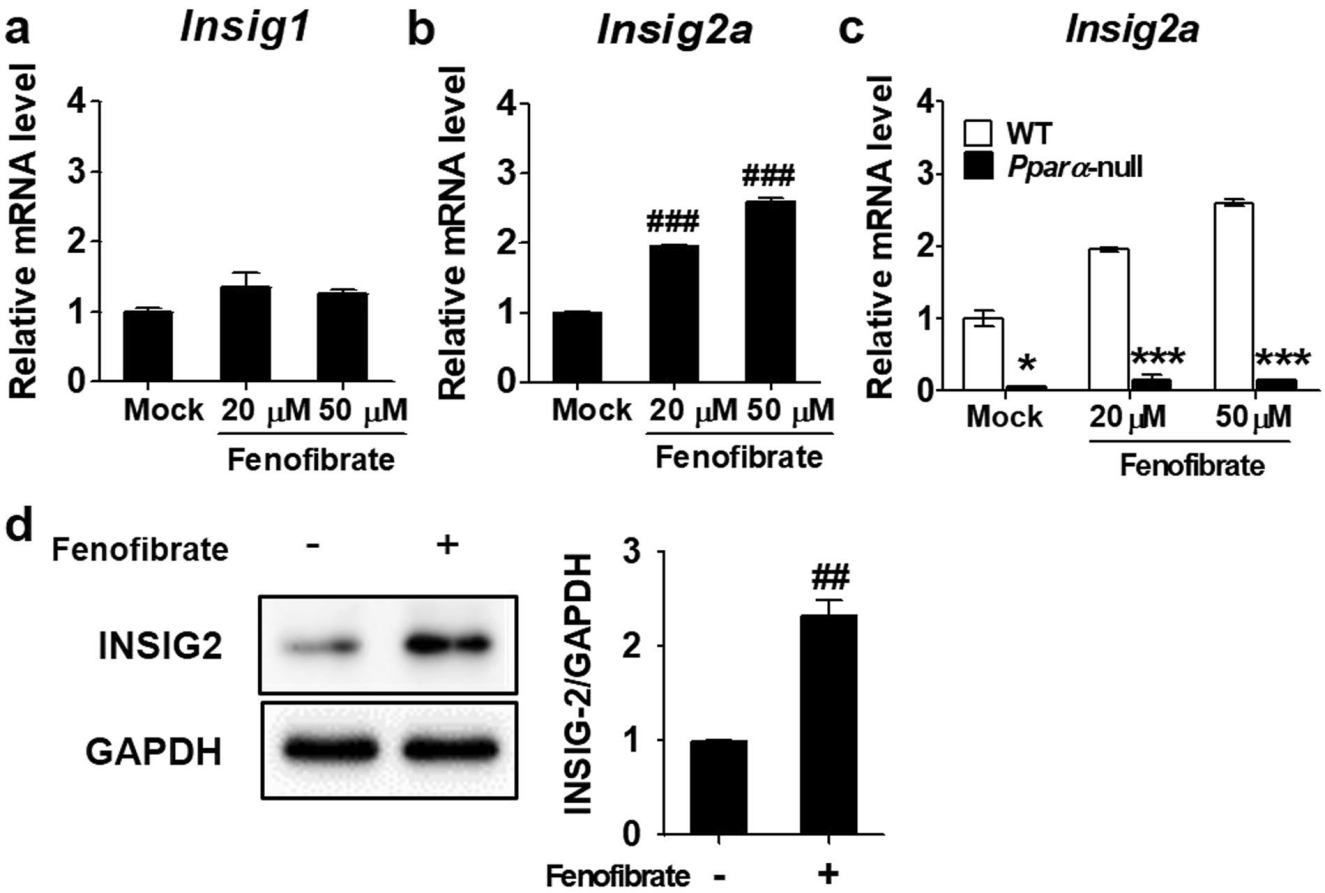
PPARα is involved in the increase of *Insig2a* gene expression. Primary hepatocytes isolated from WT mice were treated with fenofibrate at the indicated concentrations for 6 h. Total RNA was isolated and the mRNA expression levels of *Insig1* (**a**), *Insig2a* (**b**) were measured by RT-qPCR analysis. **(c)** Primary hepatocytes isolated from WT and *Pparα*-null mice were treated in the absence or presence of fenofibrate for 6 h. The expression level of *Insig2a* mRNA was measured by RT-qPCR analysis; the expression level of WT cells without treatment was regarded as 1.0. (**d**) INSIG2 protein activity was quantified by western blot analyses (cropped; full length blots can be found in Supplementary Fig. S4). The blots shown are representative of three different experiments. *p < 0.05 and ***p < 0.001 vs. WT mice. ^**##**^ *p* < 0.01 and ^**###**^ *p* < 0.001 vs. untreated group.

PPARα is a transcription factor that regulates genes required for metabolic homeostasis during fasting. To determine whether PPARα plays a role in the upregulation of *Insig2a*, primary cultured mouse hepatocytes were treated with different concentrations of fenofibrate, a PPARα agonist. The mRNA expression level of *Insig2a* was considerable increased by fenofibrate in a dose-dependent manner, whereas the *Insig1* and *Insig2b* mRNA levels were not affected by fenofibrate treatment (Fig. 2a,b, and Supplementary Fig. S2). And expression of the *Insig2a* mRNA was not significantly affected by fenofibrate in primary hepatocytes isolated from *Pparα*-null mice (Fig. 2c). Consistent with *Insig2a* mRNA expression, INSIG2 protein activity was also induced by fenofibrate treatment (Fig. 2d). These results suggest that PPARα directly upregulates *Insig2a* during fasting and is an important transcription factor for this gene.

### Defective PPARα signalling pathway does not suppress hepatic TG accumulation

**Figure 3:**
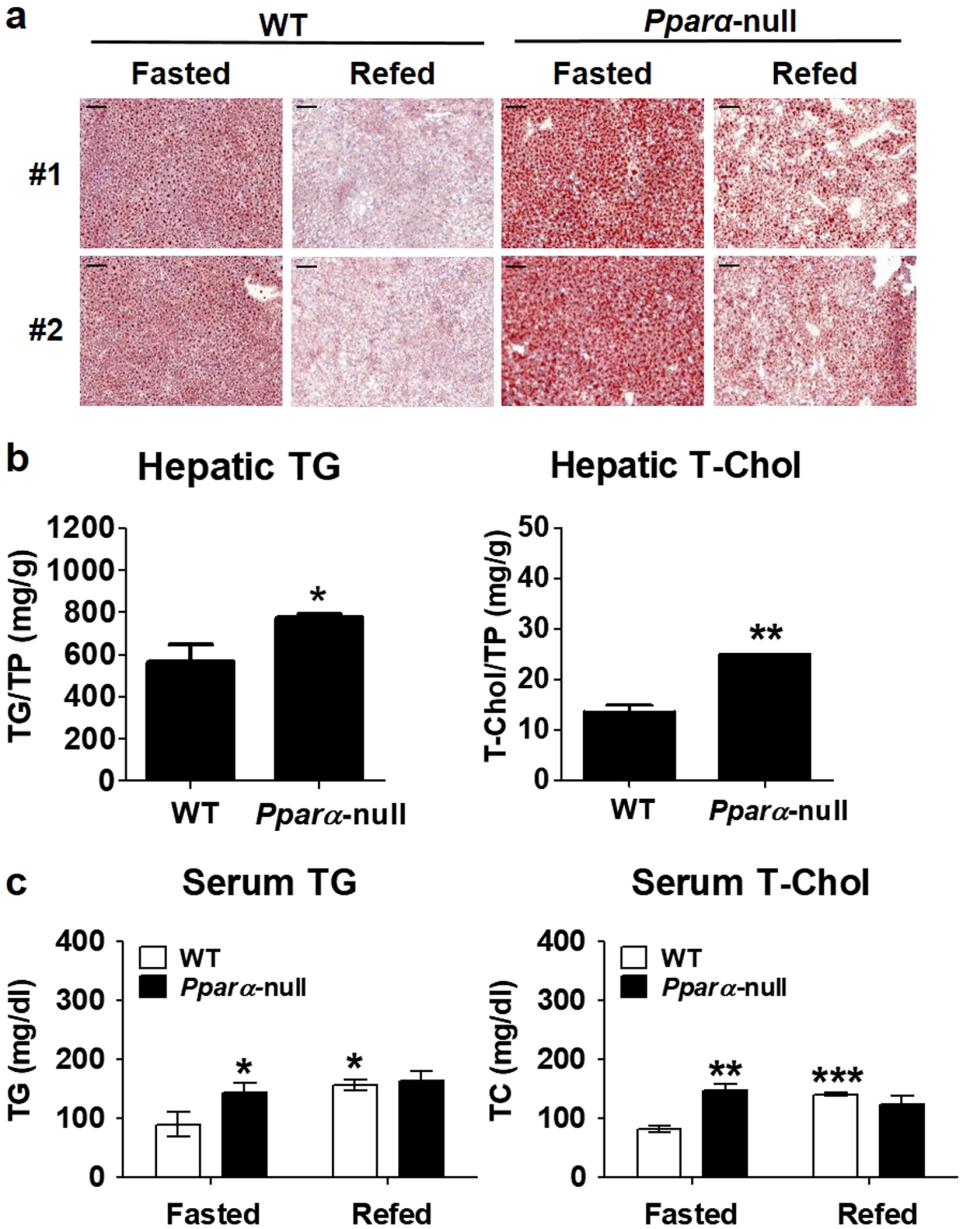
The livers of fasted *Pparα*−null mice exhibit increased lipid content. (**a**) Representative photographs of livers from WT and *Pparα*-null mice after fasting and refeeding. The livers were harvested and frozen liver sections stained with the lipid-specific oil-red O dye to reveal lipid droplets. (**b**) Hepatic triglyceride (TG) and total cholesterol in the livers of WT and *Pparα*-null mice under fasting conditions were extracted and their concentrations were determined. **(c)** Serum TG and cholesterol of WT and *Pparα*-null mice after fasting and refeeding. *p < 0.05, **p < 0.01, and ***p < 0.001 vs. WT or WT fasted mice.

*Pparα*-null mice were subjected to fasting or refeeding to assess the effects of hyperactivation of hepatic TG accumulation. There was a marked accumulation of lipid droplets in the livers of fasted *Pparα*-null mice compared with fasted wild-type (WT) mice (Fig. 3a). Consistent with the oil-red O staining results, fasting resulted in a considerable accumulation of TG and total cholesterol (T-Chol) in the livers of *Pparα*-null mice compared with WT mice (Fig. 3b). Upon further examination of the serum lipid contents, we found that serum TG and T-Chol levels were considerably higher in fasted *Pparα*-null and refed WT mice than in fasted WT mice (Fig. 3c). These results indicate that the PPARα signalling pathway is critical for inhibiting hepatic lipid homeostasis during fasting.

### PPARα-dependent *Insig2a* upregulation is mediated by fasting conditions *in vivo*

**Figure 4:**
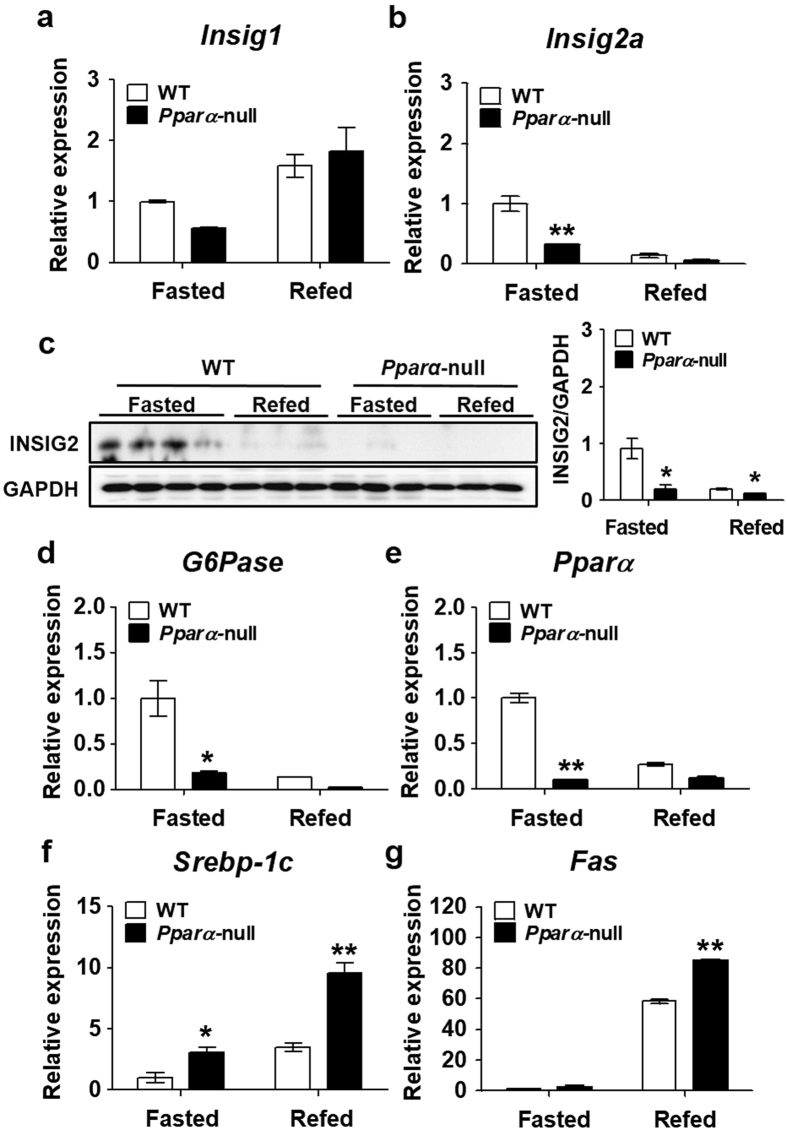
Upregulation of *Insig2a* mRNA under fasting conditions is mediated by a PPARα-dependent pathway. mRNA levels of *Insig1* (**a**), *Insig2a* (**b**) in the livers of WT and *Pparα*-null mice under fasting and refed conditions were analysed by RT-qPCR. **(c)** Protein activity of INSIG*2* in the livers of WT and *Pparα*-null mice under fasting and refed conditions. Densitometry calculations for western blot data (**c**, right panel). INSIG2 induction levels were quantified using ImageJ software (NIH) and normalised by GAPDH (cropped; full length blots can be found in Supplementary Fig. S5). mRNA levels of *G6Pase* (**d**), *Pparα* (**e**), *Srebp-1c* (**f**), and *Fas* (**g**) in the livers of WT and *Pparα*-null mice under fasted and refed conditions were measured by RT-qPCR. *p < 0.05 and **p < 0.01 vs. WT fasted mice.

The regulation of *Insig2a* by PPARα during fasting and refeeding was compared *in vivo* using WT and *Pparα*-null mice. The mRNA expression levels of *Insig1, Insig2a* and *Insig2b* were measured under fasting and refed conditions in the livers of WT and *Pparα*-null mice. *Insig1* mRNA level was increased in refed livers of both WT and *Pparα*-null mice (Fig. 4a). Change in *Insig2a* mRNA level was significantly diminished in *Pparα*-null mice during fasting compared with WT mice (Fig. 4b). Protein level of *Insig2a* under fasting conditions was markedly reduced in the livers of *Pparα*-null mice compared with those in WT mice (Fig. 4c). *﻿Insig2b* mRNA expression was higher during fasting than after refeeding in WT mice, whereas there was no difference in *Insig2b* expression between WT and Pparα-null mice during fasting (Supplementary Fig. S3﻿). G*6Pase*, a known target gene of PPARα, exhibited changes similar to those observed for *Insig2a* and *Pparα* in *Pparα*-null mice (Fig. 4d,e). In contrast, the expression levels of SREBP-1c and its target gene *Fas* were increased in refed WT mice, and were further increased in *Pparα*-null mice (Fig. 4f,g). These results suggest that upregulation of *Insig2a* during fasting was mediated by a PPARα**-**dependent pathway.

### Insulin-stimulated SREBP-1c processing is suppressed by a PPARα agonist

**Figure 5:**
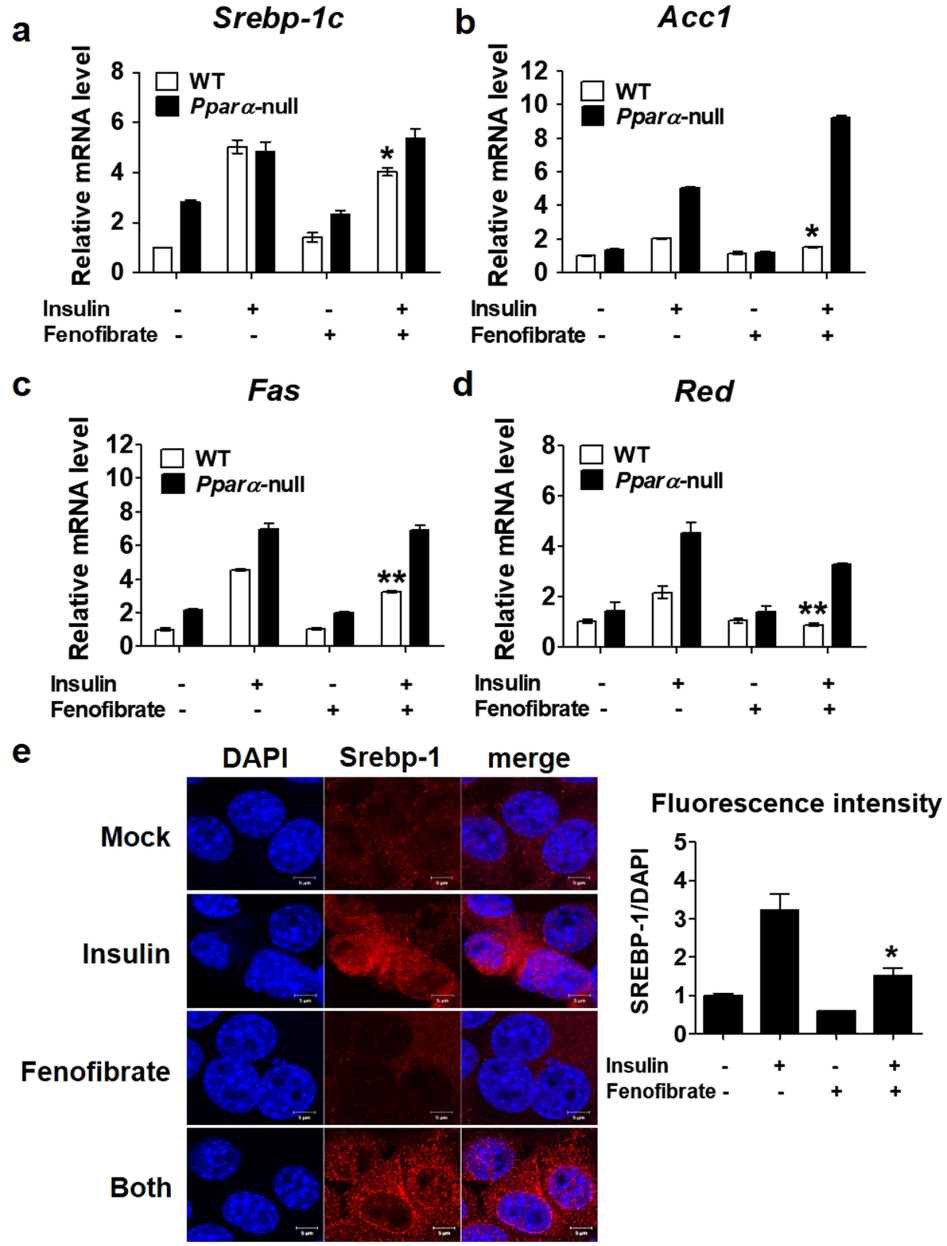
PPARα suppresses insulin-induced activation of lipogenic gene expression. mRNA levels of *Srebp-1c* (**a**), *Acc1* (**b**), *Fas* (**c**), and *Red* (**d**) in primary hepatocytes extracted from WT and *Pparα*-null mice. Cells were incubated with 1 µM insulin and/or 50 µM fenofibrate for 6 h and analysed by RT-qPCR. (**e**) Fenofibrate suppresses insulin-induced nuclear localization of SREBP-1. AML12 cells were incubated with 1 µM insulin and/or 50 µM fenofibrate for 2 h; translocation of SREBP-1 was determined using immunofluorescence by conforcal laser microscopy and then was analysed by LSM 3 EXCITER software. Fluorescence intensity for immunofluorescence data (e, right panel). *p < 0.05 and **p < 0.01 vs. the insulin-treated group.

To investigate whether PPARα activation could inhibit insulin-mediated induction of lipogenic genes, primary hepatocytes from WT and *Pparα*-null mice were treated with fenofibrate in the presence of insulin. mRNA expression levels of *Srebp-1c* and lipogenic genes such as *Acc1, Fas*, and *Red* were higher in *Pparα*-null hepatocytes than in WT hepatocytes (Fig. 5a,b,c,d). In the hepatocytes of *Ppar*α-null mice, the expression of insulin-induced lipogenic genes increased considerably and the inhibitory effects of fenofibrate were diminished. These results demonstrated that PPARα could negatively regulate lipogenic genes. Furthermore, inhibition of nuclear translocation of SREBP-1c by PPARα was also confirmed in AML12 cells by immunocytochemistry. As shown in Fig. 5e, insulin induced SREBP-1c activity and increased its nuclear translocation, whereas insulin coupled with fenofibrate treatment resulted in reduced nuclear translocation despite increased SREBP-1c activity in the ER. This result indicates that activation of PPARα could inhibit SREBP-1c processing, thus inhibiting the translocation of SREBP-1c to the nucleus.

### Knockdown of *Insig2a* enhances SREBP-1c processing

**Figure 6:**
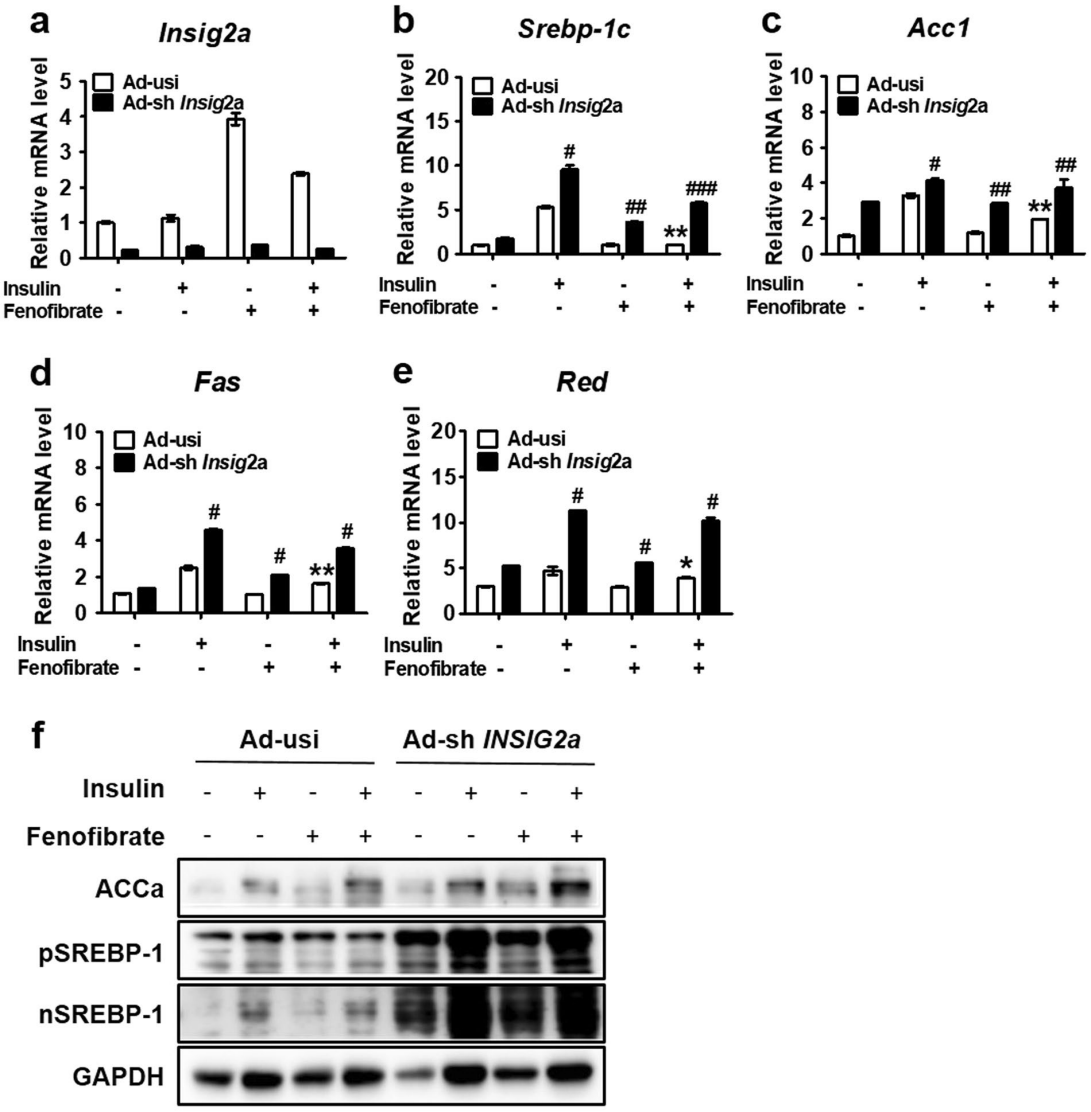
Fenofibrate regulates insulin-induced hepatic lipogenic gene expression via *Insig2a*. AML12 cells were infected with adenovirus (Ad)-sh*Insig2a* or Ad-usi as an unspecific RNAi for 48 h and incubated with 1 μM insulin and/or 50 μM fenofibrate for 6 h. Total RNA was collected and mRNA expressions of *Insig2a* (**a**), *Srebp-1c* (**b**), *Acc1* (**c**), *Fas* (**d**), and *Red* (**e**) genes were analysed by RT-qPCR. (**f**) Whole cell lysates were prepared from AML12 cells infected with Ad-sh*Insig2a*, followed by treatment with 1 μM insulin and/or 50 μM fenofibrate for 24 h, and were analysed by immunoblotting with indicated antibodies (cropped; full length blots can be found in Supplementary Fig. S6). *p < 0.05 and **p < 0.01 vs. the insulin-treated group. ^#^ *p* < 0.05, ^##^ *p* < 0.01 and ^###^ *p* < 0.05 vs. the Ad-usi-treated group.

To determine whether PPARα inhibits insulin-mediated lipogenic gene upregulation through an *Insig2a*-dependent mechanism, *Insig2a* was depleted from AML12 cells using short hairpin RNA (shRNA) followed by treatment with insulin and/or fenofibrate. AML12 cells were infected with unspecific RNAi adenovirus and sh*Insig2a* adenovirus for 48 hrs, and cells overexpressed with sh*Insig2a* adenovirus showed a 90% reduction in *Insig2a* mRNA levels compared with control cells (Fig. 6a). The mRNA levels of *Srebp-1c* and its target genes such as *Acc1*, *Fas*, and *Red* were increased in the presence of insulin; the addition of fenofibrate resulted in decreased expression. Cells infected with sh*Insig2a* adenovirus exhibited higher basal mRNA levels of those genes; insulin treatment further increased expression to higher levels than those observed in the control virus-infected cells (Fig. 6b,c,d,e). In addition to the increase in mRNA levels, the precursor and mature forms of SREBP-1c and its target protein also showed increased activity upon insulin treatment in sh*Insig2a* virus-infected cells (Fig. 6f). Taken together, these results suggest that PPARα is a key mediator of repression of hepatic lipogenesis-related genes through the upregulation of *Insig2a* in AML12 cells.

### PPARα binds the PPRE on the mouse *Insig2a* promoter during fasting

**Figure 7:**
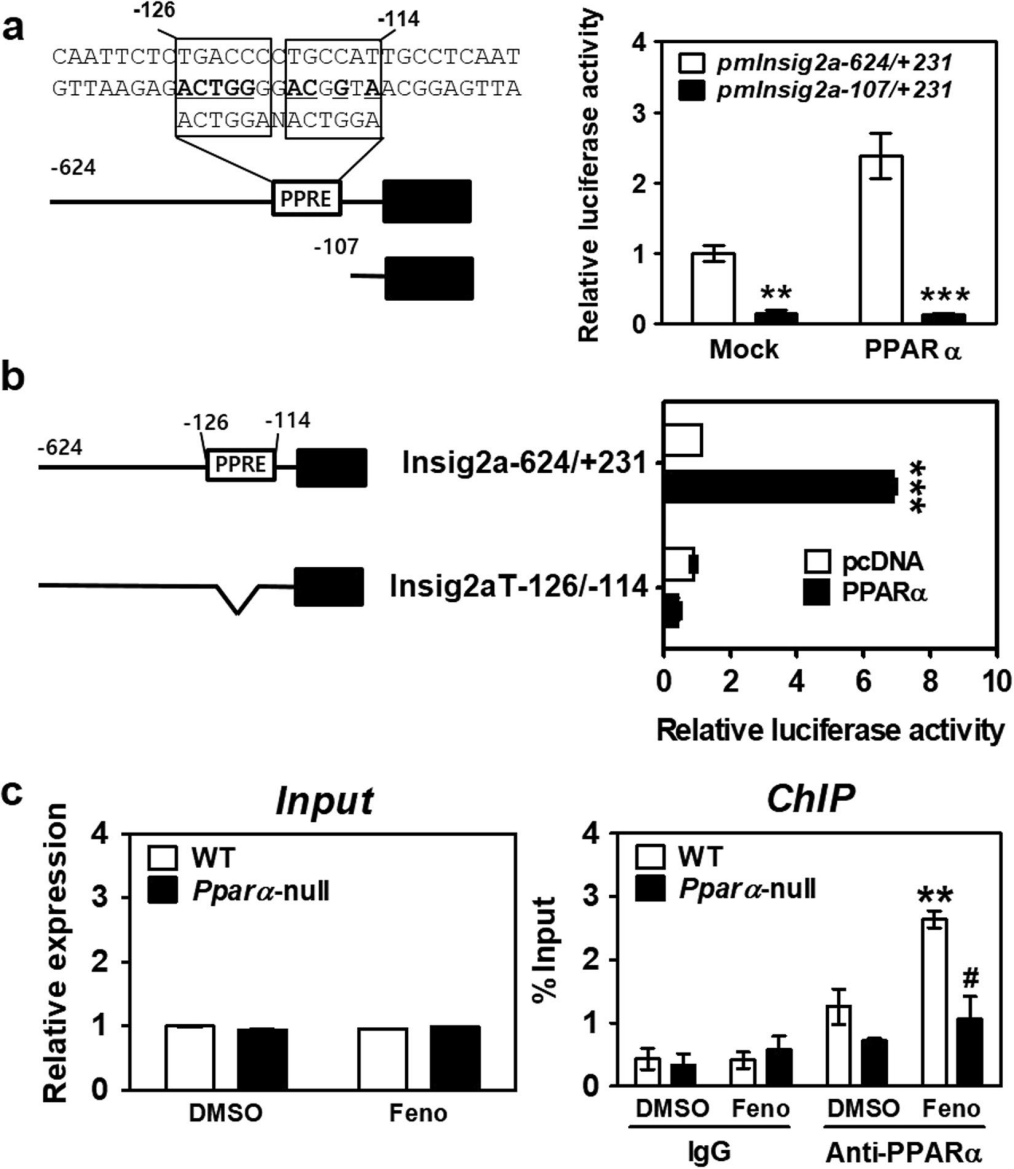
PPRE in the mouse *Insig2a* promoter is responsive to PPARα. (**a**) Effects of PPARα on promoter activity of the *Insig2a* gene. The proposed PPARα binding element is located between −126 bp and −114 bp from the transcription start site (boxed). Plasmids that contained the full *Insig2a* promoter (open bars) or the basal promoter region where the putative PPRE was deleted (filled bars) were co-transfected with protamine complementary DNA (pcDNA) or a PPARα expression vector in HEK293T cells and treated with 50 μM fenofibrate. The transfection efficiency of each sample was normalised to β-galactosidase activity. (**b**) Plasmid constructs with an internal deletion of the PPRE in the full *Insig2a* promoter were used for a luciferase assay. (**c**) Chromatin immunoprecipitation (ChIP) assays using chromatin isolated from the livers of WT and *Pparα-*null mice were performed. The input represents 10% purified DNA for each sample. Nuclear extracts from the livers of WT and *Pparα*-null mice were immunoprecipitated with an anti-PPARα antibody, and purified DNA was subjected to qPCR using primers specific for the PPRE region of the mouse *Insig2a* gene promoter. All data are representative of at least three independent experiments. *p < 0.05, **p < 0.01 and ***p < 0.001 vs. untreated or WT control. ^#^ *p* < 0.05 vs. WT fenofibrate-treated group.

To better understand how PPARα regulates *Insig2a* gene transcription, consensus PPRE sequences on the promoter of the mouse *Insig2a* (m*Insig2a*) gene were identified; a highly conserved PPRE sequence was identified between −126 bp and −114 bp from the transcription start site (Fig. 7a). Plasmids containing various lengths of the promoter region of mouse *Insig2a* were cloned and transfected into HEK293T cells. Plasmids containing the full-length promoter showed the highest response to fenofibrate treatment; deletion up to −107 bp markedly diminished this response (Fig. 7a). Moreover, the deletion of the putative PPRE between −126 bp and −114 bp from the full-length m*Insig2a* sequence resulted in decreased promoter activity upon fenofibrate treatment (Fig. 7b). These results indicate that the predicted PPRE, located between −126 bp and −114 bp of the m*Insig2a* gene promoter, is responsive to PPARα. The binding of PPARα to this PPRE was further verified at the chromatin level by a ChIP assay using mouse liver tissue (Fig. 7c). These results implied that *Insig2a* gene expression is regulated through the direct binding of PPARα to the *Insig2a* promoter during fasting.

## Discussion

We showed that the expression of *Insig2a*, a target gene of PPARα, increased during fasting and decreased during refeeding in the livers of WT mice, along with parallel changes in PPARα expression. Therefore, nutritional status should be a critical factor for investigating the physiological functions of PPARα. Previous studies that showed the elevation of fatty acid synthesis by PPARα agonists measured the mRNA levels of *Pparα* and *Insig2a* without considering the effects on SREBP-1c^15, 16^. Our study showed that decreased *Insig2a* expression led to an increase in SREBP-1c expression in *Pparα*-null mice during fasting.

Fenofibrate, a PPARα agonist, is currently used to lower lipid levels in clinical practice; its fundamental mechanism is to suppress PPARα-mediated activation of fatty acid oxidation^17^. Previous studies have demonstrated that PPARα agonists inhibit SREBP-1c activity and, thereby, TG synthesis^18^. This was consistent with our findings that fenofibrate inhibited SREBP-1c processing in hepatocytes, resulting in the inhibition of the expression of fatty acid synthesis genes; this effect was diminished in *Pparα*-null mice. Even though PPARα ligands could be used to combat hepatic steatosis in non-alcoholic fatty liver disease (NAFLD), it is evident that PPARα ligands reduce liver fat in rodents^19, 20^, but not necessarily in humans^21^. Furthermore, we identified a PPRE on the mouse *Insig2a* gene promoter, suggesting that PPARα may regulate the processing of the cleaved-activated form of SREBP-1 via upregulation of *Insig2a* gene expression (Fig. 6). These data raise the possibility of a cross-talk between PPARα and the lipogenic transcription factor, SREBP-1c.

INSIG1 and INSIG2 are important regulators of SREBP processing which can influence the rates of TG and cholesterol synthesis. This implies that their activity might be associated with human metabolic disorders. Similarly, functional variations within the human *Insig2* promoter seem to affect the body mass index (BMI)^22^. Both INSIG1 and INSIG2 can bind to the SCAP/SREBP complex and cause its retention in the ER in a sterol-dependent manner, even though they are reciprocally regulated after feeding. While *Insig1* expression is regulated by SREBPs under refed conditions, liver-specific *Insig2a* expression is induced by fasting^16^. However, the specific roles of each INSIG protein in the regulation of metabolism are not clear and remain to be discovered.

**Figure 8:**
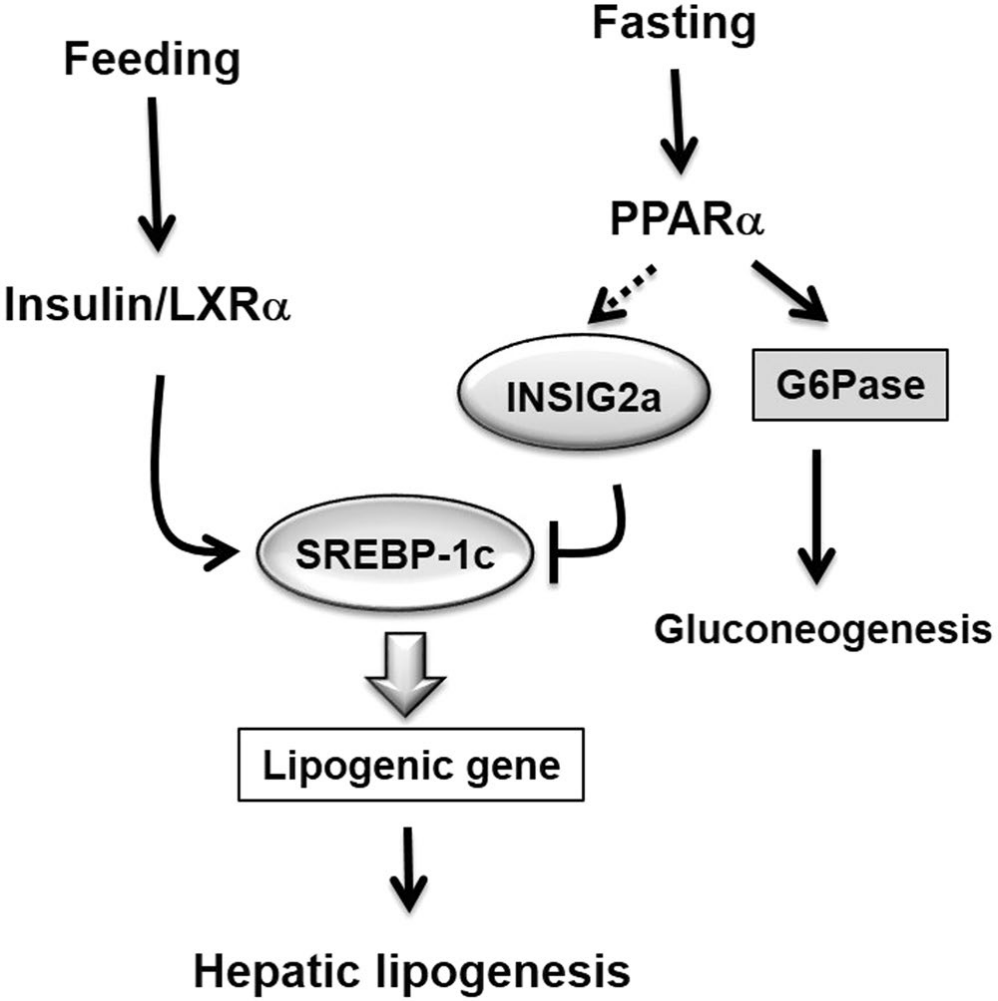
Proposed mechanism of the regulation of SREBP-1c by PPARα through Insig2a in the liver.

Here, we identified that PPRE responds to PPARα by binding to the mouse *Insig2a* promoter in the region between −126 bp and −114 bp. The direct binding of PPARα to this site was confirmed by a ChIP assay. These results strongly indicate that PPARα is a direct activator of the *Insig2a* promoter, and therefore might be involved in the regulation of SREBP processing (Fig. 8). This also indicates that fenofibrate could ameliorate fat accumulation by repressing hepatic lipogenesis through the upregulation of *Insig2a*. Although our findings may currently lack clinical relevance, our data have demonstrated that the PPARα/INSIG2 interaction is related to the downregulation of the hepatic lipogenic pathway during fasting, showing a logical and corresponding physiological effect with potential applications for future medical interventions. Therefore, it will be necessary to further clarify this signalling pathway in diseased animal models.

## Materials and Methods

### Reagents

Fenofibrate was purchased from Sigma-Aldrich (St. Louis, MO) and dissolved in DMSO. Antibodies against ACC1 and FAS were purchased from Cell Signaling Technology (Danvers, MA). The SREBP-1 antibody which used for the ChIP assay and immunoblotting and anti-β-actin were purchased from Santa Cruz Technology (Santa Cruz Biotechnology, Dallas, TX) and anti-SREBP-1 used for immunocytochemistry was kindly gifted by Dr. Timothy F. Osborne (Sanford Burnham Prebys Medical Discovery Institute, Orlando, FL). Adenoviruse expressing sh*Insig-2a* was a kind gift from Dr. Baoliang Song (College of Life Sciences, Wuhan University, Wuhan, China).

### Animal studies

Male C57BL6 mice were obtained from Jung-Ang Experimental Animals (Seoul, Republic of Korea). *Pparα*-null mice were studied at 8 weeks of age as described previously^23^. Fasted mice were deprived of food for 24 h, followed by refeeding for 12 h with a high carbohydrate diet (D12079B; Research Diets Inc., New Brunswick, NJ, USA). All animal procedures and care administered were approved by the Institutional Animal Use and Care Committee (IAUCC), Keimyung University School of Medicine (KM-2012–49R and KM-2014–34R3). All experiments were carried out in accordance with the approved guidelines.

### Isolation and culture of primary mouse hepatocytes

Mouse primary hepatocytes were isolated from WT and *Pparα*-null mice as described previously^24^. Briefly, isolated primary hepatocytes were cultured in Dulbecco’s modified Eagle’s medium (DMEM; Invitrogen, Carlsbad, CA, USA) containing 10% heat-inactivated FBS, 100 units/ml penicillin G, 100 μg/ml streptomycin, 10 μM dexamethasone, 100 nM insulin, and 25 mM glucose. Cells were incubated in DMEM containing 25 mM glucose, 0.5% BSA, 100 units/ml penicillin G, and 100 μg/ml streptomycin for 16 h before the experiment. And cells were treated with 50 μM fenofibrate which was purchased from Sigma-Aldrich (St. Louis, MO, USA) and dissolved in DMSO.

### Cell culture

AML-12 immortalised mouse hepatocytes (CRL-2254; ATCC, Manassas, VA, USA) were cultured in DMEM/F-12 medium (GIBCO-BRL, Gaithersburg, MD, USA) supplemented with 10% FBS, insulin-transferrin-selenium (GIBCO-BRL), 40 ng/ml dexamethasone (Sigma-Aldrich, St. Louis, MO, USA), 100 nM insulin, and antibiotics in a humidified atmosphere containing 5% CO_2_ at 37 °C.

### qPCR analysis

Total RNA was isolated from the livers or primary hepatocytes of mice using the TRIzol method (Invitrogen, Carlsbad, CA, USA). First-strand cDNA synthesis was performed using the cDNA superscript kit (Bio-Rad, Hercules, CA, USA), which was then analysed by qPCR using the CFX96 Real-Time PCR system (Bio-Rad). All data were normalised against the expression level of ribosomal L32. The following primer sets were used: *Insig1*: forward, 5′-TCACAGTGACTGAGCTTCAGCA-3′; reverse, 5′-TCATCTTCATCACACCCAGGAC-3′; *Insig2a*: forward, 5′-CCCTCAATGAATGTACTGAAGGATT-3′; reverse, 5′-TGTGAAGTGAAGCAGACCAATGT-3′; *Insig2b*: forward, 5′-CCGGGCAGAGCTCAGGAT-3′; reverse, 5′-GAAGCAGACCAATGTTTCAATGG-3′; *Pparα*: forward, 5′-AGAGCCCCATCTGTCCTCTC-3′; reverse, 5′-ACTGGTAGTCTGCAAAACCAAA-3′; *Srebp-1c*: forward, 5′-GGAGCCATGGATTGCACATT-3′; reverse, 5′-GGCCCGGGAAGTCACTGT-3′; *Fas*: forward, 5′-GCTGCGGAAACTTCAGGAAAT-3′; reverse, 5′-AGAGACGTGTCACTCCTGGACTT-3′; *Acc1a*: forward, 5′-TGACAGACTGATCGCAGAGAAAG-3′; reverse, 5′-TGGAGAGCCCCACACACA-3′; *RED*: forward, 5′-CTTGTGGAATGCCTTGTGATTG-3′; reverse, 5′-AGCCGAAGCAGCACATGAT-3′; *G6Pase*: forward, 5′-CGACTCGCTATCTCCAAGTGA-3′; reverse, 5′-GTTGAACCAGTCTCCGACCA-3′; *L32*: forward, 5′-ACATTTGCCCTGAATGTGGT-3′; reverse, 5′-ATCCTCTTGCCCTGATCCTT-3′.

### Immunoblotting

Proteins were prepared and immunoblotting was performed as described previously^24^. Proteins from the cells and tissues were separated by SDS-PAGE and transferred onto nitrocellulose membranes. The membranes were incubated with antibodies against ACC1 and FAS (Cell Signaling Technology), SREBP-1 kindly provided by Dr. Timothy F. Osborne (Sanford Burnham Prebys Medical Discovery Institute, Orlando, FL, USA), and β-actin (Santa Cruz Biotechnology), and developed using an enhanced chemiluminescent western blot detection kit (Amersham Bioscience, Piscataway, NJ, USA). Band intensities below the saturation threshold were measured using ImageJ software. Values are expressed as the integrals of each band.

### Construction of plasmids, transfection of cells, and luciferase assays

The promoters containing the regions of −624/+231 and −107/+231 bp of mouse *Insig2a* were synthesised by PCR, inserted into the pGL3basic vector (Promega, Madison, WI, USA), and designated pm*Insig2a* (−624/+231) and pm*Insig2a* (−107/+231). The internal deletion mutant of the PPRE site (−126/−114) was constructed from pm*Insig2a* (−624/+231) using a PPRE-deletion primer (sense, 5′-GCCAATTCTCTGCCTCAATAAATGCTTGC-3′; antisense, 5′-TATTGAGGCAGAGAATTGGCAGAGCTAA-3′. HEK239T cells were plated in 12-well plates at a density of 5 × 10^4^ cells/well in 1 ml medium. The plasmids containing the promoter regions (0.2 µg), pCMV-β-galactosidase (100 ng), and the murine PPARα expression vector (0 or 100 ng) were transfected into cells as described previously^25^. Media containing the indicated concentrations of fenofibrate were added to the cells. After 24 h, cells were harvested and luciferase assays were performed as described previously^25^.

### Oil-red O staining of cryosections

After fasting and refeeding, liver tissues were embedded in Tissue-Tek OCT Compound (Sakura Japan Co., Ltd., Tokyo, Japan), and 5-μm sections were mounted on slides and stored at −80 °C. Before staining with oil-red O, slides were dried at 25 °C and fixed in 10% formalin for 10 min at 4 °C. After fixation, slides were dried and immersed in 60% isopropyl alcohol, and then stained with oil-red O for 30 min at room temperature. Slides were briefly destained in 60% isopropyl alcohol and counterstained with haematoxylin.

### TG and cholesterol measurement in liver

Livers were homogenised using a tissue Lyzer (BD Bioscience, Franklin Lakes, NJ, USA) after adding 1 ml physiologic saline. The solution was centrifuged, and the supernatant was used for quantification of TG levels. Total TG and cholesterol concentrations were measured by a Beckman Coulter AU480 automatic biochemistry analysis system (Model AU-480). The average TG concentration was calculated in the liver by dividing the total mean values by the total protein contents.

### ChIP assay

A ChIP assay was performed as described previously^26^. Briefly, mouse primary hepatocytes were isolated from WT and *Pparα-*null mice. The cells were incubated with 50 µM fenofibrate for 24 h. The cells were then incubated with paraformaldehyde for 15 min and subjected to a ChIP assay using anti-PPARα. Each sample contained 25 ng chromatin. The extracted DNA from the final step was quantified by PCR with primers specific for the putative PPRE region (−126 bp/−114 bp) of the *Insig2a* promoter. The raw *C*_*t*_ values of the ChIP samples were divided by the *C*_*t*_ values of the relevant input samples and the values were presented as a percentage of the input values (% input). The specific primers used for PCR are as follows: mouse *Insig2a*: forward, 5′-TCACATCAGGGGACAGTTAG-3′; reverse, 5′-TAAGCAAATAGAGAACTCCC-3′; mouse *Gapdh*: forward, 5′-CCTGGAGAAACCTGCCAAGTA-3′; reverse, 5′-TGGAAGAGTGGGAGTTGCTGT-3′.

### Immunocytochemistry

AML12 cells were plated on 8-chamber culture dishes and cultured in control medium or media containing insulin (1 μM) and/or fenofibrate (50 μM). After incubation for 2 h, the cells were fixed with 4% paraformaldehyde in PBS. The fixed cells were permeabilised and then incubated in blocking solution (1% BSA and 0.1% Triton-X in PBS) at room temperature for 1 h, followed by incubation with anti-SREBP-1 primary antibody diluted in blocking solution at 4 °C overnight. The cells were incubated with Alexa Fluor-conjugated secondary antibodies (Life Technologies, Grand Island, NY, USA) for 1 h at room temperature. The chamber slides were sealed with fluorescent mounting medium containing DAPI (Molecular Probes, Eugene, OR, USA). Images were acquired by confocal laser scanning microscopy (Carl Zeiss, Thornwood, NY, USA).

### Statistical analysis

All data are shown as mean ± standard deviation (SD). Statistical differences between groups were evaluated by the Student’s *t* test, one-way analysis of variance (ANOVA), or two-way ANOVA using GraphPad Prism 5.0 software. *p* < 0.05 was considered statistically significant.

## Electronic supplementary material

Supplementary information

## References

[1] Nohturfft A, DeBose-Boyd RA, Scheek S, Goldstein JL, Brown MS (1999). Sterols regulate cycling of SREBP cleavage-activating protein (SCAP) between endoplasmic reticulum and Golgi. Proc Natl Acad Sci U S A.

[2] Foretz M (1999). ADD1/SREBP-1c is required in the activation of hepatic lipogenic gene expression by glucose. Mol Cell Biol.

[3] Yabe D, Brown MS, Goldstein JL (2002). Insig-2, a second endoplasmic reticulum protein that binds SCAP and blocks export of sterol regulatory element-binding proteins. Proc Natl Acad Sci U S A.

[4] Engelking LJ (2005). Schoenheimer effect explained–feedback regulation of cholesterol synthesis in mice mediated by Insig proteins. J Clin Invest.

[5] Yabe D, Komuro R, Liang G, Goldstein JL, Brown MS (2003). Liver-specific mRNA for Insig-2 down-regulated by insulin: implications for fatty acid synthesis. Proc Natl Acad Sci U S A.

[6] Engelking LJ (2004). Overexpression of Insig-1 in the livers of transgenic mice inhibits SREBP processing and reduces insulin-stimulated lipogenesis. J Clin Invest.

[7] Yellaturu CR, Deng X, Park EA, Raghow R, Elam MB (2009). Insulin enhances the biogenesis of nuclear sterol regulatory element-binding protein (SREBP)-1c by posttranscriptional down-regulation of Insig2a and its dissociation from SREBP cleavage-activating protein (SCAP).SREBP-1c complex. J Biol Chem.

[8] Braissant O, Foufelle F, Scotto C, Dauca M, Wahli W (1996). Differential expression of peroxisome proliferator-activated receptors (PPARs): tissue distribution of PPAR-alpha, -beta, and -gamma in the adult rat. Endocrinology.

[9] Issemann I, Green S (1990). Activation of a member of the steroid hormone receptor superfamily by peroxisome proliferators. Nature.

[10] Dreyer C (1993). Positive regulation of the peroxisomal beta-oxidation pathway by fatty acids through activation of peroxisome proliferator-activated receptors (PPAR). Biol Cell.

[11] Kersten S (1999). Peroxisome proliferator-activated receptor alpha mediates the adaptive response to fasting. J Clin Invest.

[12] Abdelmegeed MA (2011). PPARalpha expression protects male mice from high fat-induced nonalcoholic fatty liver. J Nutr.

[13] Boergesen M (2012). Genome-wide profiling of liver X receptor, retinoid X receptor, and peroxisome proliferator-activated receptor alpha in mouse liver reveals extensive sharing of binding sites. Mol Cell Biol.

[14] Seo YK (2009). Genome-wide analysis of SREBP-1 binding in mouse liver chromatin reveals a preference for promoter proximal binding to a new motif. Proc Natl Acad Sci U S A.

[15] Knight BL (2005). A role for PPARalpha in the control of SREBP activity and lipid synthesis in the liver. Biochem J.

[16] Nohturfft A, Zhang SC (2009). Coordination of lipid metabolism in membrane biogenesis. Annu Rev Cell Dev Biol.

[17] Staels B, Maes M, Zambon A (2008). Fibrates and future PPARalpha agonists in the treatment of cardiovascular disease. Nat Clin Pract Cardiovasc Med.

[18] Konig B (2009). Activation of PPARalpha and PPARgamma reduces triacylglycerol synthesis in rat hepatoma cells by reduction of nuclear SREBP-1. Eur J Pharmacol.

[19] Staels B (2013). Hepatoprotective effects of the dual peroxisome proliferator-activated receptor alpha/delta agonist, GFT505, in rodent models of nonalcoholic fatty liver disease/nonalcoholic steatohepatitis. Hepatology.

[20] Ip E, Farrell G, Hall P, Robertson G, Leclercq I (2004). Administration of the potent PPARalpha agonist, Wy-14,643, reverses nutritional fibrosis and steatohepatitis in mice. Hepatology.

[21] Fernandez-Miranda C (2008). A pilot trial of fenofibrate for the treatment of non-alcoholic fatty liver disease. Dig Liver Dis.

[22] Krapivner S (2008). Insulin-induced gene 2 involvement in human adipocyte metabolism and body weight regulation. J Clin Endocrinol Metab.

[23] Im SS (2011). Peroxisome proliferator-activated receptor {alpha} is responsible for the up-regulation of hepatic glucose-6-phosphatase gene expression in fasting and db/db Mice. J Biol Chem.

[24] Kang HS (2016). Metformin stimulates IGFBP-2 gene expression through PPARalpha in diabetic states. Sci Rep.

[25] Im SS (2005). Glucose-stimulated upregulation of GLUT2 gene is mediated by sterol response element-binding protein-1c in the hepatocytes. Diabetes.

[26] Im SS (2011). Linking lipid metabolism to the innate immune response in macrophages through sterol regulatory element binding protein-1a. Cell Metab.

