# Supplementary material for: PPARα-dependent Insig2a overexpression inhibits SREBP-1c processing during fasting
Source: Sci Rep. 2017 Aug 30;7:9958. doi: 10.1038/s41598-017-10523-7 (PMC5577246; doi:10.1038/s41598-017-10523-7)

**<Supplementary information>**

**PPARα-dependent INSIG2a overexpression inhibits SREBP-1c processing during fasting**

Jae-Ho Lee^1^, Hye Suk Kang^1^, Hyeon Young Park^1^, Young-Ah Moon^2^, Yu Na Kang^3^, Byung-Chul Oh^4^, Dae-Kyu Song^1^, Jae-Hoon Bae^1^, and Seung-Soon Im^1^,*

^1^Department of Physiology, Keimyung University School of Medicine, Daegu 42601, South Korea.

^2^Department of Molecular Medicine, Inha University School of Medicine, Incheon 22212, South Korea.

^3^Department of Pathology, Keimyung University School of Medicine, Daegu 42601, South Korea.

^4^Lee Gil Ya Cancer and Diabetes Institute, College of Medicine, Gachon University, Incheon, 21999, Korea; Department of Physiology, College of Medicine, Gachon University, Incheon 21999, South Korea.

*S. S. Im (PhD), Department of Physiology, Keimyung University School of Medicine, Daegu 42601, South Korea. Tel.: 82-53-580-3863; Fax: 82-53-580-3793; E-mail: ssim73@kmu.ac.kr

**Supplementary Fig. S1. Full-length images of the immunoblots in Figure 1.** Black dot line boxes indicate the cropped images used in Figure 1.


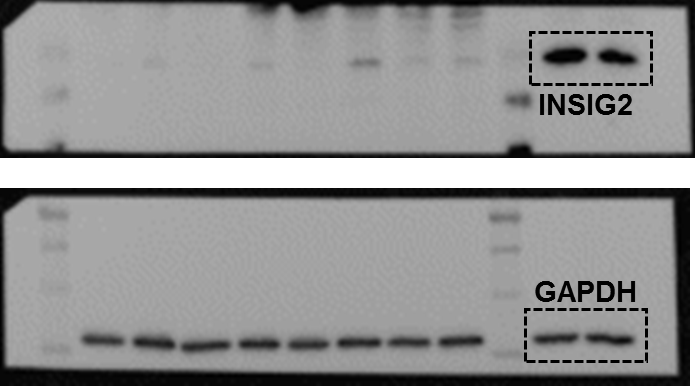


**Supplementary Fig. S2. *Insig2b* gene expression.** Primary hepatocytes extracted from WT mice. Total RNA was isolated and the mRNA expression level of *Insig2b* was measured by RT-qPCR analysis.

**Supplementary Fig. S3. *Insig2b* gene expression.** mRNA levels of Insig2b in the livers of WT and Pparα-null mice under fasting and refed conditions were analysed by RT-qPCR. **p < 0.01 compared between WT fasted mice and WT refed mice.

.

**Supplementary Fig. S4. Full-length images of the immunoblots in Figure 2.** Black dot line boxes indicate the cropped images used in Figure 2.


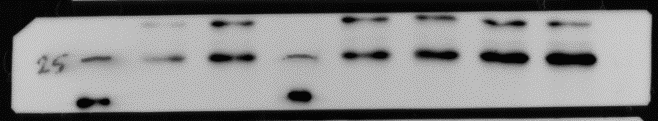


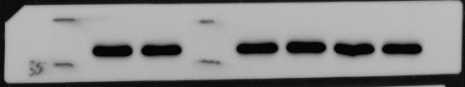


**INSIG2**

**GAPDH**

**Supplementary Fig. S5. Full-length images of the immunoblots in Figure 4.** Black dot line boxes indicate the cropped images used in Figure 4.

**
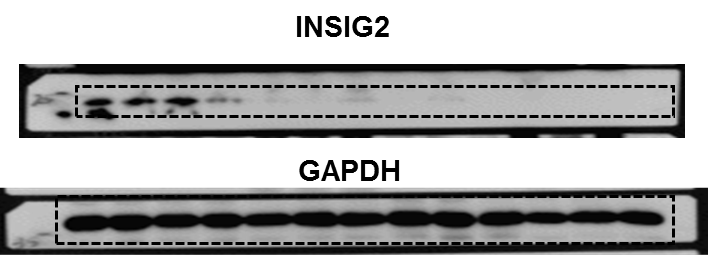
**

**Supplementary Fig. S6. Full-length images of the immunoblots in Figure 6.** Black dot line boxes indicate the cropped images used in Figure 6.


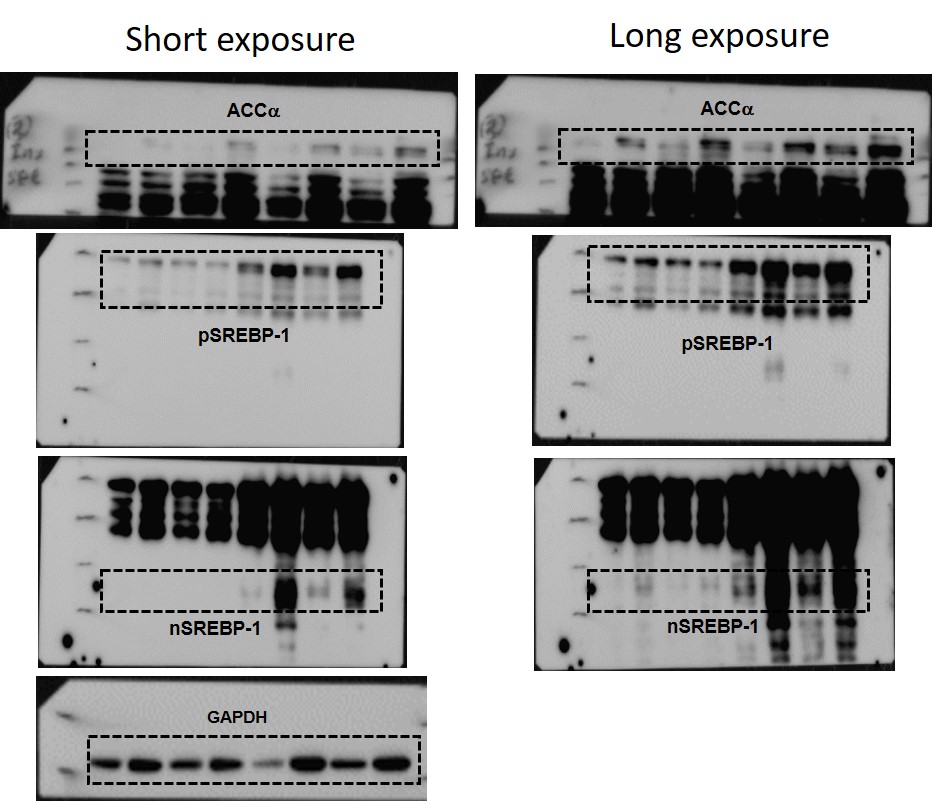

Supplement: Supplementary file 1 — Supplementary information [file 41598_2017_10523_MOESM1_ESM.doc]
